# Effects of co-application of tiotropium bromide and traditional Chinese medicine on patients with stable chronic obstructive pulmonary disease: a muilticenter, randomized, controlled trial study

**DOI:** 10.3389/fmed.2024.1289928

**Published:** 2024-05-03

**Authors:** Ruilin Chen, Kaiwen Ni, Conghua Ji, Zhongda Liu, Yali Yu, Gang Liu, Junchao Yang, Zhen Wang

**Affiliations:** ^1^The First Clinical College of Zhejiang Chinese Medical University, Zhejiang Chinese Medical University, Hangzhou, China; ^2^The First Affiliated Hospital of Zhejiang Chinese Medical University, Zhejiang Chinese Medical University (Zhejiang Provincial Hospital of Chinese Medicine), Hangzhou, China; ^3^College of Public Health, Zhejiang Chinese Medical University, Hangzhou, China; ^4^Lishui Hospital of TCM, Lishui, China; ^5^Ningbo Municipal Hospital of TCM, Ningbo, China; ^6^Wenzhou TCM hospital of Zhejiang Chinese medical university, Wenzhou, China

**Keywords:** chronic obstructive pulmonary disease, RCT study, clinical trial, traditional Chinese medicine, quality of life

## Abstract

**Background:**

Chronic Obstructive Pulmonary Disease (COPD) is a common, preventable, and treatable disease. Traditional Chinese Medicine (TCM) has shown promising potential in COPD treatment. and we conducted a multi-center RCT to evaluate the effectiveness of TCM-based therapy in stable COPD patients.

**Methods:**

In this multicenter, double-blind RCT, a total of 200 patients were supposed to be assigned to either trial or control group randomly. Both groups received Tiotropium (18 μg) from month 0 to month 12. Trial group received additional TCM granules, while control group received a placebo from month 0 to month 6. Symptom assessment, total effective rate, lung function measurements, hospitalization rates, and quality of life were evaluated at month 0, month 6, and month 12. Adverse events were assessed at month 12.

**Results:**

Of the initial 105 patients (aged 40–80) who completed the study, 51 were in trial group and 54 were in control group. At month 6, significant differences were observed between two groups in total effective rate (*p* = 0.020), sputum score (*p* = 0.047), changes in FVC% (*p* = 0.047) and FEV1 (*p* = 0.046). At month 12, significant differences were observed in sputum score (*p* = 0.020), FVC (*p* = 0.042), and change in FEV1 (*p* = 0.013). Compared to baseline, they both demonstrated improvements in symptoms, acute exacerbation, lung function, quality of life, and exercise tolerance.

**Conclusion:**

TCM treatment effectively improved total effective rate, sputum symptom, FVC%, FEV1, and exhibited prolonged efficacy in improving sputum symptoms and FEV1 in stable COPD patients.

**Clinical trial registration:**https://www.chictr.org.cn/showproj.html?proj=6029 identifier ChiCTR-TRC-13003531.

## Introduction

Chronic Obstructive Pulmonary Disease (COPD) is a common, preventable, and treatable disease characterized by persistent respiratory symptoms and airflow limitation caused by airway and/or alveolar abnormalities (1). Over the period from 1970 to 2002, the mortality rate attributed to COPD doubled, highlighting the significance of this condition (2). It is recognized as a major cause of morbidity and mortality globally, leading to a substantial social and economic burden (3, 4). In light of these concerns, we initiated this trial. According to the World Health Organization (WHO), COPD is currently the third leading cause of death, accounting for approximately 6% of total deaths in 2019 (5). In China alone, it was estimated that around 99.9 million individuals were affected by COPD (6). The burden of morbidity and mortality associated with COPD remains a pressing issue.

The Global Initiative for Chronic Obstructive Lung Disease (GOLD) (1) emphasizes that the main treatment goals for stable COPD are the reduction of symptoms and the prevention of future exacerbations. Tiotropium bromide, a maintenance therapy for stable COPD (7), has demonstrated significant benefits in terms of reducing symptoms such as dyspnea, slowing lung function decline, decreasing exacerbation rates, and improving quality of life (8). Furthermore, an increasing body of evidence supports the unique advantages of traditional Chinese medicine (TCM) in clinical practice. The combination of TCM with conventional Western medicine has shown significant differences in acute exacerbation frequency, duration, symptoms, 6-min walking distance (6MWT), and dyspnea scale (9). A clinical trial has also indicated that Jinshui Liujun decoction has significant clinical efficacy in the treatment of severe and extremely severe COPD in the elderly. It can effectively alleviate clinical symptoms, reduce the number of acute exacerbations, alleviate inflammatory reactions, and improve quality of life (10). A meta-analysis implied that the CAT score, mMRC and average hospitalization time were also reduced significantly by Shengmai injection plus western medicine (11).

Moreover, experimental studies exploring the underlying pathological mechanisms have revealed the potential benefits of TCM in COPD. For instance, One network pharmacology indicated that Jinshui Liujun decoction can treat COPD through the synergy of multiple ingredients, multiple targets and multiple pathways (12). However, limited multi-center randomized controlled trials (RCTs) have been conducted to validate the efficacy of TCM in the treatment of stable COPD. Therefore, we have designed an RCT to evaluate the effectiveness of comprehensive therapy based on a specific TCM pattern in stable COPD patients. Traditional Chinese herbs used in this trial, as a combination of Jinshui Liujun decoction and Shengmai decoction, consist of Danggui [Dry root of Angelica sinensis (Oliv.) Diels.], Shudi (Dried root of Rehmannia glutinosa Libosch.), Chenpi (Dried mature pericarp of *Citrus reticulata* Blanco.), Jiangbanxia [Underground tubers of pinellia ternate (Thunb.) Breit processed with ginger and alum.], Fuling [Sclerotium of Poria cocos (Schw.) Wolf.], Zhigancao (Dry roots and rhizomes of Glycyrrhiza uralensis Fisch, Glycyrrhiza inflata Bat, Glycyrrhiza qlabra L.), Taizishen [Dry root tubers of Pseudostellaria heterophylla (Miq.) Pax ex Pax et Hoffm.], Tiandong [root tuber of Asparagus cochinchinensis (Lour.) Merr.], Maidong (Fleshy root of *Ophiopogon japonicus* Ker-Gawl.), Wuweizi (Matured fruits of Schisandra chinensis Baill.), Huangqi [Dried root of Astraqalus membranaceus Bge. Var. mongholicus (Bge.) Hsiao, Astraqalus membranaceus (Fisch.) Bge.]

## Materials and methods

### Ethical approval

The research protocol underwent a comprehensive review and received approval from the Ethics Committee of the First Affiliated Hospital of Zhejiang Chinese Medical University, with the approval identifier 2012-K-001-02. Prior to the start of the trial, all participants were provided with clear explanations of the trial procedures, and written informed consent was obtained from each individual.

### Subjects and experimental paradigm

The study enrolled stable COPD patients with a diagnosis of qi-yin deficiency syndrome of the lung and kidney (Table 1) from January 2013 to December 2015. Patients were recruited from the respiratory outpatient departments of five tertiary hospitals in Zhejiang province. All patients met the diagnostic criteria for group B to D in the stable stage of COPD. A multicenter, randomized, double-blind, controlled clinical trial was conducted, and a total of 200 patients were divided equally into the control group (*n* = 100) and the experimental group (*n* = 100).

**Table 1:** Criteria of syndrome of qi-yin deficiency of lung and kidney.

Syndrome of qi-yin deficiency of lung and kidney	Symptoms
Satisfied with at least 2/3 items	(1) Shortness of breath, exacerbated by exercise;
	(2) Sweating or fatigued, deteriorated by exercise;
	(3) Susceptible;
Satisfied with at least 2/5 items	(4) Soreness and weakness of waist and knees;
	(5) Tinnitus or vertigo;
	(6) Cough with or without sputum which is tough to be spat out;
	(7) Perspire during sleeping;
	(8) Feverish sensation over the palm and sole;
Required option	(9) Body of the tongue is off-colour or brightly red, the appearance of sunken and thin pulse or thin and weak pulse;

## Eligible criteria and exclusion criteria

### Eligible criteria

(1) Patients who met the diagnostic criteria for COPD in the stable stage according to GOLD (2010) (7). (2) Age between 40 and 80 years. (3) Patients diagnosed with qi-yin deficiency syndrome of the lung and kidney (criteria listed in Table 1). (4) Willingness to provide informed consent and signed informed consent form.

### Exclusion criteria

(1) Patients with severe underlying diseases affecting the liver, kidney, brain, or hematopoietic system; (2) Patients with a diagnosis of tumor, hyperthyroidism, diabetes, or peptic ulcer; (3) Pregnant or lactating women; (4) Patients with psychiatric disorders; (5) Patients with lower limb dysfunction; (6) Patients currently participating in other clinical trials; (7) Patients unwilling to receive the prescribed interventions or unable to cooperate due to other reasons.

### Sample size

The sample size for this study was calculated using the superiority test formula (1) with a 1:1 ratio between the trial group and the control group.

*P_c_* represented the alleviation rate of control group. Based on our research, *P_c_* = 0.73, *P_T_* represented the alleviation rate of trial group, and we set *P_T_* = 0.88. α, set as 0.05, means type I error. Thus, *Z_α_* = 1.645. β, as type II error, equals to 0.20, therefore, *Z_β_* = 0.842. considering a 20% drop of this research, the final sample size is 100 patients in each group.

(1) $$n=\frac{{\left(Z_{\alpha }+Z_{\beta }\right)}^{2}\left[P_{C}\left(1-P_{C}\right)+P_{T}\left(1-P_{T}\right)\right]}{{\left(P_{T}-P_{C}\right)}^{2}}$$

### Randomization and intervention

This design adopts a randomized double-blind controlled trial, in which patients are divided into the experimental group and the control group using a random number table. Participants were randomly assigned in a 1:1 ratio to either the control group or the trial group using a random number generator. At the same time, primary and secondary blinds are set up, and blinding is performed by non-participating staff. All direct participants in the trial are unaware of the blinding until unblinding is completed.

During the first 6 months, the control group received tiotropium bromide 18ug once daily, along with a placebo of traditional Chinese medicine. The placebo of TCM consisted of dextrin, bitters, food coloring, 5% medication. The control group received one dose per day. On the other hand, the trial group received tiotropium bromide 18ug once daily, along with traditional Chinese medicine granules which consist of Danggui, Shudi, Chenpi, Jiangbanxia, Fuling, Zhigancao, Taizishen, Tiandong, Maidong, Wuweizi, Huangqi. Both placebo and traditional Chinese medicine granules produced under GMP conditions by Jiang Yin Tian Jiang Pharmaceutical Co., Ltd. The trial group also received one dose per day. In the second 6 months, both the control group and the trial group received tiotropium bromide 18ug once daily. Throughout the one-year observation period, occurrences of acute exacerbations of chronic obstructive pulmonary disease (AECOPD) were monitored, and patients received immediate medical care when necessary.

### Safety assessment

Any functional damage will be assessed during one-year observation period. If any severe safety events, such as respiratory failure or other serious complications confirmed by clinicians occur, experimental intervention will be stopped immediately, and proper treatment should be provided.

### Outcomes

The primary outcome is the total effective rate, which includes the scores of cough, shortness of breath, sputum, tiredness, loose stool and reduced appetite. The total effective rate is calculated using formula (2). These symptoms were assessed at baseline (month 0), at the sixth month (month 6), and at the twelfth month (month 12).

The secondary outcomes are as follows. Acute exacerbation of COPD (AECOPD) is characterized by an increase in symptoms of dyspnea, cough, sputum volume, and sputum purulence, accompanied by increased airway inflammation, mucus hypersecretion, and gas trapping (13). The evaluation of AECOPD includes the number of acute attacks, duration and frequency of hospitalization at month 6 and month 12. Differences in lung function parameters were also analyzed, including forced vital capacity (FVC), percentage of predicted FVC (FVC%), forced expiratory volume in 1 s (FEV1), percentage of predicted FEV1 (FEV1%), and FEV1/FVC ratio. The dyspnea scale questionnaire (mMRC) (14), the 6-min walking distance test (6MWT) (15), and the COPD Assessment Test (CAT) (16) were applied. Lung function data, mMRC scores, and 6MWT results were obtained at month 0, month 6, and month 12. Safety analysis, including the assessment of adverse events, was conducted at month 12.

(2) $$EI=\frac{\mathrm{Scor}e_{\mathrm{before}-\mathrm{treatment}}-\mathrm{Scor}e_{\mathrm{after}-\mathrm{treatment}}}{\mathrm{Scor}e_{\mathrm{before}-\mathrm{treatment}}}\times 100\%$$

### Statistical analysis

All *p*-values were calculated using a two-tailed test, and the significance level (α) was set at 0.05. First, a normality test was conducted to assess the distribution of the data. For variables that exhibited a normal distribution, the results were presented as mea*n* ± standard deviation (SD). The comparison between the trial group and the control group (age, BMI, symptoms, 6MWT, lung function, CAT) was analyzed using Student’s t-test. Paired sample t-tests were employed to evaluate the differences before and after the intervention for variables such as symptoms, 6MWT, and lung function. Classification variables, including gender and smoking status, were analyzed using the Chi-square test, and the results were expressed as number (percentage). Variables that did not conform to a normal distribution were represented as median (minimum, maximum), and the comparison between groups was conducted using the Kruskal-Wallis test for variables such as mMRC, acute attack, and hospitalization. Paired Kruskal-Wallis tests were utilized to examine the differences before and after the intervention for the mMRC variable. Covariate analysis of variance (ANOVA) was employed for variables with uneven baseline distributions, such as FVC and FEV1. Statistical analyses were performed using IBM SPSS 22.0 for Windows and SAS 9.2.

## Results and discussion

### Comparison of general information

**Figure 1:**
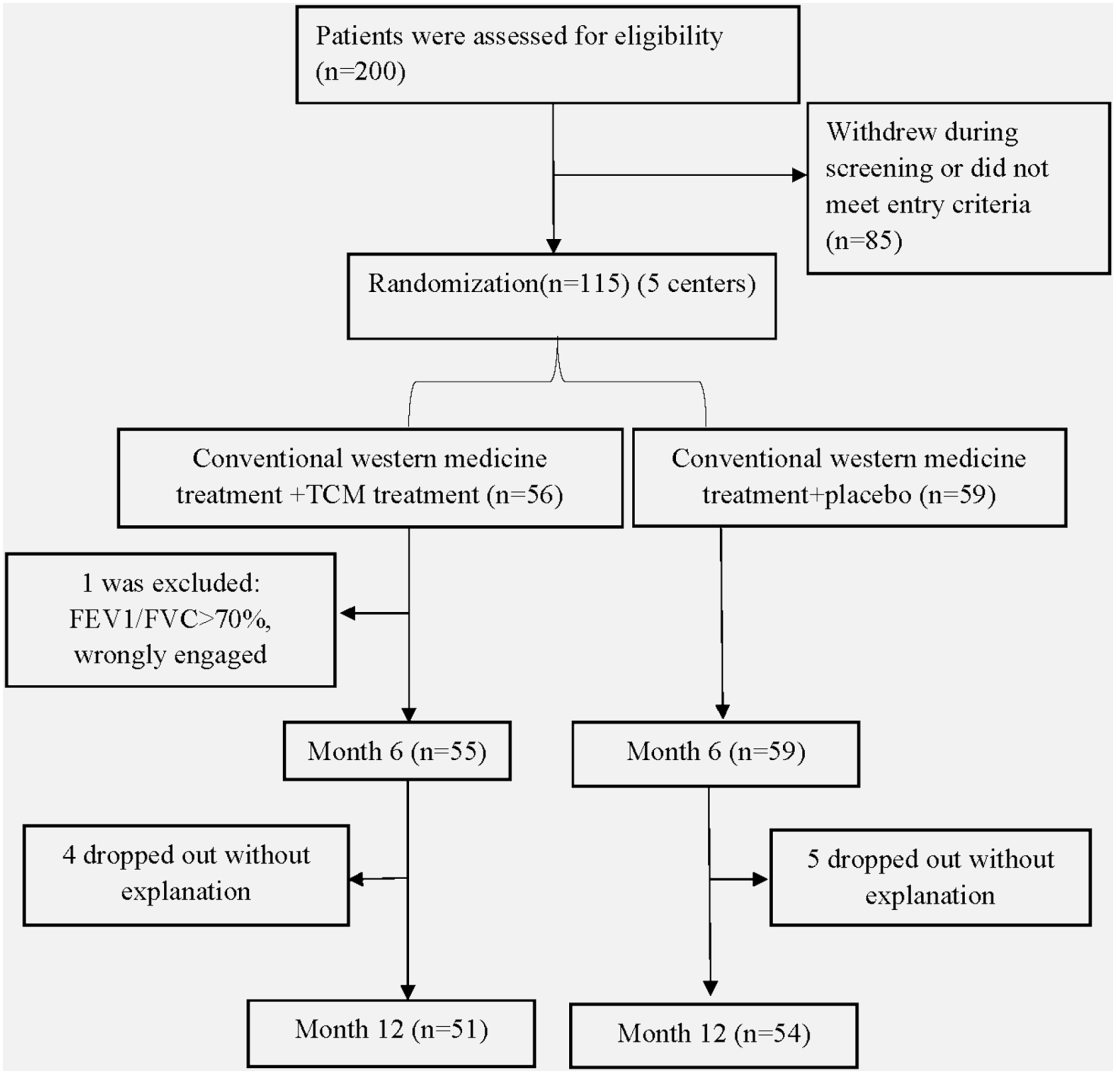
Enrollment of patients and completion of the subgroup analysis.

A total of 200 patients were initially recruited for the study. After applying the withdrawal and exclusion criteria, 115 patients were eligible and agreed to participate in the trial. Out of these, 114 patients remained in the study until the 6th month, and ultimately, 105 patients successfully completed the entire trial (Figure 1). The 114 patients were randomly assigned to either the control group (*n* = 59) or the trial group (*n* = 55). There were no significant differences observed between the two groups in terms of general characteristics (age, gender), symptoms (shortness of breath, cough, and sputum), hospitalization frequency and duration, BMI, smoking status, lung function (FVC%, FEV1%, FEV1/FVC), exercise tolerance (6MWT, mMRC), and quality of life (CAT). Table 2 provides a detailed overview of the general information for both groups.

**Table 2:** Baseline characteristics of the patients.

Characteristics	Trial group (*n* = 55)	Control group (*n* = 59)	total (*n* = 114)	Group comparison	
				t/χ^2^/z	*p*
Age (year)	70.65 ± 8.07	67.78 ± 8.59	69.17 ± 8.43	*t* = 1.84	0.069
Gender				*χ*^2^ = 0.18	0.675
Male *n* (%)	44 (80.00)	49 (83.05)	93 (81.58)		
Female *n* (%)	11 (20.00)	10 (16.95)	21 (18.42)		
BMI^※^	23.01 ± 3.03	22.81 ± 3.38	22.91 ± 3.20	*t* = 0.33	0.743
Smoking status				*χ*^2^ = 0.118	0.731
Not smoking *n* (%)	26 (47.27)	26 (44.07)	52 (45.61)		
Currently smoking *n* (%)	29 (52.73)	33 (55.93)	62 (54.39)		
Symptoms					
Shortness of breath	4.47 ± 1.90	4.17 ± 1.67	4.32 ± 1.79	*t* = 0.90	0.368
Cough	2.44 ± 1.13	2.17 ± 0.85	2.30 ± 1.00	*t* = 1.41	0.161
Sputum	2.33 ± 1.20	2.34 ± 0.99	2.33 ± 1.09	*t* = 0.06	0.955
Acute attack					
Frequency (times)	1.00 (0.00,7.00)	1.00 (0.00, 9.00)	1.00 (0.00, 9.00)	Z = 0.318	0.751
Hospitalization ^□^					
Frequency (times)	1.00 (0.00, 4.00)	0.00 (0.00, 9.00)	1.00 (0.00, 9.00)	Z = 1.079	0.281
Duration (day)	10.00 (0.00, 65.00)	0.00 (0.00, 63.00)	9.00 (0.00, 65.00)	Z = 0.969	0.333
Lung function					
FVC	2.09 ± 0.64	2.40 ± 0.80	2.25 ± 0.74	*t* = 2.25	0.026
FVC%	64.46 ± 15.08	68.37 ± 17.66	66.48 ± 16.51	*t* = 1.27	0.208
FEV1	1.07 ± 0.34	1.31 ± 0.53	1.20 ± 0.46	*t* = 2.91	0.004
FEV1%	43.94 ± 13.51	47.52 ± 15.90	45.79 ± 14.84	*t* = 1.29	0.200
FEV1/FVC	52.34 ± 10.25	54.65 ± 10.63	53.54 ± 10.47	*t* = 1.18	0.240
CAT	16.36 ± 6.01	15.58 ± 5.83	15.96 ± 5.91	*t* = 0.71	0.480
6MWT	365.89 ± 115.81	373.14 ± 116.81	369.64 ± 115.87	*t* = 0.33	0.740
mMRC				*Z* = 0.969	0.333
0 *n* (%)	3 (5.45)	4 (6.78)	7 (6.14)		
1 *n* (%)	14 (25.45)	23 (38.98)	37 (32.46)		
2 *n* (%)	30 (54.55)	18 (30.51)	48 (42.11)		
3 *n* (%)	8 (14.55)	13 (22.03)	21 (18.42)		
4 *n* (%)	0 (0.00)	1 (1.69)	1 (0.88)		

^※^BMI = weight (Kg)/height^2^(m). ^□^Hospitalizations within the 12-month period before screening were self-reported.

### Comparison of total effective rate

At month 6, a significant difference was observed in the total effective rate between the trial group and the control group (*p* < 0.05). However, by month 12, the effectiveness of TCM granule treatment had diminished, and there was no significant difference between the two groups (*p* = 0.216, Table 3).

**Table 3:** Comparison of total effectiveness.

Visiting points		Trial group	Control group	Group comparison	
				*χ* ^2^	*p*
Month 6	Effective (EI ≥ 30%), *n* (%)	21 (38.18%)	11 (18.64%)	5.382	0.020*
	Invalid (EI<30%), *n* (%)	34 (61.82%)	48 (81.36%)		
Month 12	Effective (EI ≥ 30%), *n* (%)	21 (41.18%)	16 (29.63%)	1.532	0.216
	Invalid (EI<30%), *n* (%)	30 (58.82%)	38 (70.37%)		

*p < 0.05, compared to control group.

### Comparison of symptoms of shortness of breath, cough, and sputum

**Figure 2:**
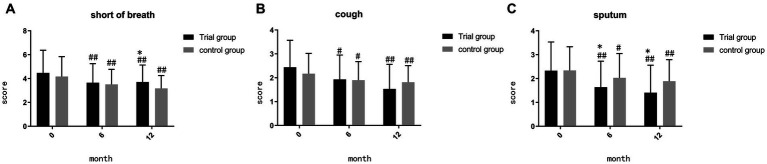
**(A)** comparison of score of shortness of breath during month 0, 6, 12; **(B)** comparison of score of cough during month 0, 6, 12; **(C)** comparison of score of sputum during month 0, 6, 12; ##*p* < 0.01, compared to baseline, #*p* < 0.05, compared to baseline, **p < 0.01, compared to control group, *p < 0.05, compared to control group.

Before the initiation of treatment, no significant differences were found between the two groups in terms of shortness of breath, cough, and sputum (*p* = 0.368, *p* = 0.161, *p* = 0.955). During month 6, the trial group exhibited a notable reduction in sputum symptoms compared to the control group (*p* = 0.047) (Figure 2C). By month 12, the trial group demonstrated superiority in sputum reduction compared to the control group (*p* = 0.020) (Figure 2C), but it showed inferiority in reducing shortness of breath (*p* = 0.03) (Figure 2A). Both the trial group and the control group exhibited significant effectiveness compared to baseline (*p* < 0.05).

### Comparison of the frequency of acute attack and hospitalization, duration of hospitalization

**Figure 3:**
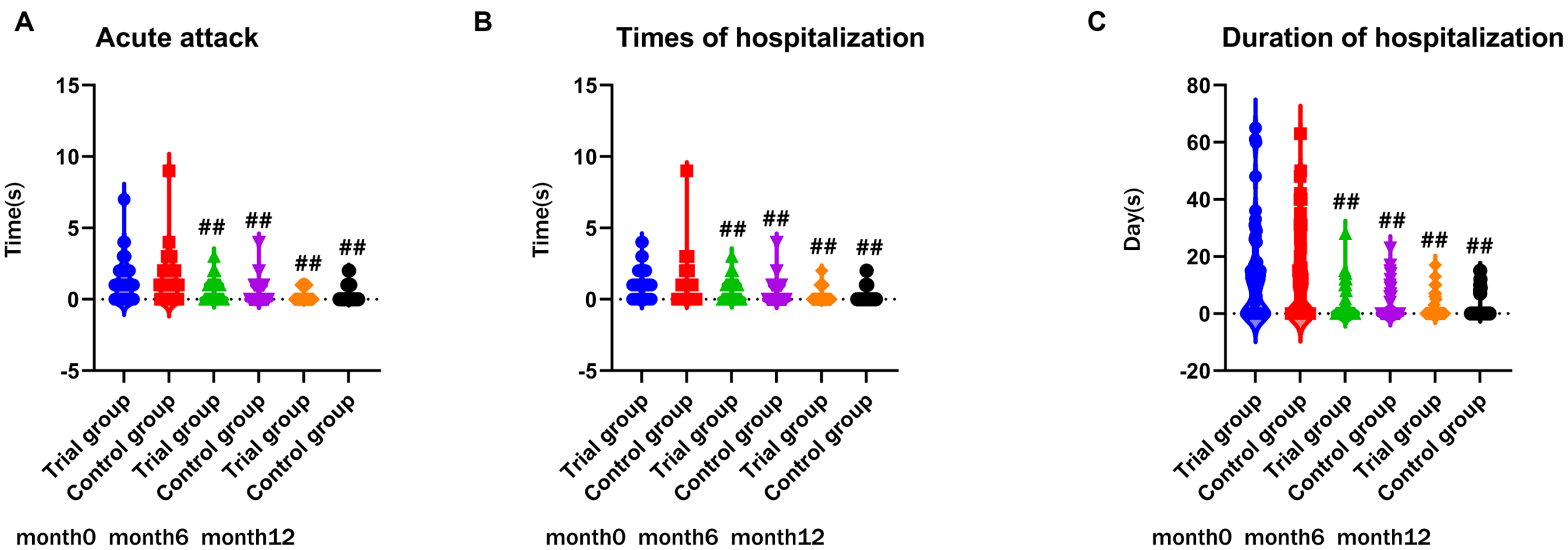
**(A)** comparison of frequency of acute attack during month 0, 6, 12; **(B)** comparison of frequency of hospitalization during month0, 6, 12; **(C)** comparison of duration of hosipitalization during month 0, 6, 12; ##*p* < 0.01, compared to baseline, #*p* < 0.05, compared to baseline, **p < 0.01, compared to control group, *p < 0.05, compared to control group.

Prior to treatment, there were no significant differences observed in exacerbation rates between the two groups (*p* = 0.751, *p* = 0.281, *p* = 0.333, Figure 3). Throughout the one-year observation period, no significant differences were found when comparing the two groups. As depicted in Figure 3, both the trial group and the control group exhibited a significant reduction in the frequency and duration of hospitalizations compared to baseline (*p* < 0.01).

### Comparison of lung function

**Figure 4:**
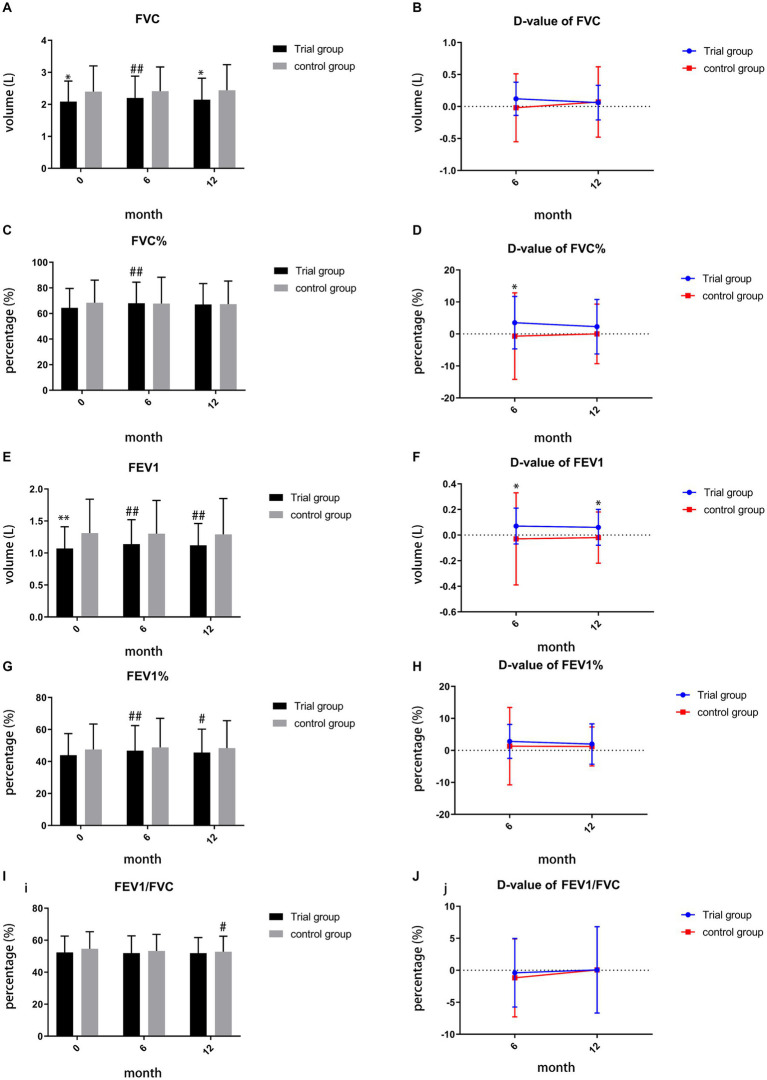
**(A)** comparison of FVC during month 0, 6, 12; **(B)** comparison of D-value of FVC during month 6, 12; **(C)** comparison of FVC% during month0, 6,12; **(D)** comparison of D-value of FVC% during month 6, 12; **(E)** comparison of FEV1 during month 0, 6, 12; **(F)** comparison of D-value of FEV1 during month 6, 12; **(G)** comparison of FEV1% during month 0, 6, 12; **(H)** comparison of D-value of FEV1% during month 6, 12; **(I)** comparison of FEV1/FVC during month 0, 6, 12; **(J)** comparison of D-value of FEV1/FVC during month 6,12; ##*p* < 0.01, compared to baseline, #*p* < 0.05, compared to baseline, **p < 0.01, compared to control group, *p < 0.05, compared to control group.

Compared to baseline, TCM demonstrated its superiority in improving FVC, FVC%, FEV1, and FEV1% during month 6 (*p* = 0.001, *p* = 0.002, *p* < 0.001, *p* < 0.001, Figures 4A,C,E,G). By month 12, TCM exhibited a significant difference in improving FEV1 (*p* = 0.003, Figure 4E) and FEV1% (*p* = 0.029, Figure 4G), while the control group showed decline in FEV1/FVC (*p* = 0.044, Figure 4I). However, during month 12, the trial group demonstrated inferiority in improving FVC compared to the control group (*p* = 0.042, Figure 4A). We also calculated the difference value (D-value) between the finish-line and baseline measurements for comparing the two groups. During month 6, the D-value of FVC% and FEV1 in the trial group suggested a significant difference compared to the control group (*p* = 0.047, *p* = 0.046, Figures 4D,F). By month 12, TCM maintained its superiority in the D-value of FEV1 (*p* = 0.013, Figure 4F) and showed no significant difference in the D-value of FVC (*p* = 0.936, Figure 4B).

### Comparison of quality of life (CAT) and exercise tolerance

**Figure 5:**
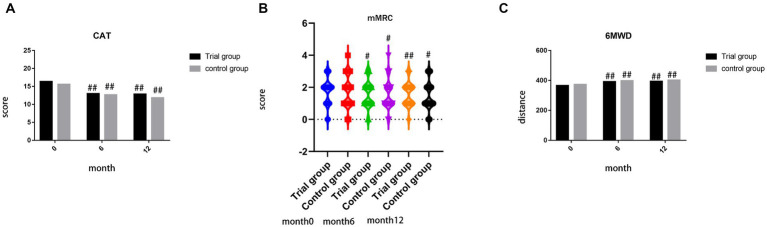
**(A)** comparison of score of CAT during month 0, 6, 12; **(B)** comparison of score of mMRC during month 0, 6, 12; **(C)** comparison of distance of 6MWT during month 0, 6, 12; ##*p* < 0.01, compared to baseline, #*p* < 0.05, compared to baseline, **p < 0.01, compared to control group, *p < 0.05, compared to control group.

There were no significant differences observed in the scores of CAT, mMRC, and 6MWT prior to treatment (*p* = 0.480, *p* = 0.333, *p* = 0.740). Furthermore, no significant differences were found in the scores of CAT, mMRC, and 6MWT between the trial group and control group during month 6 and month 12. However, both the trial group and control group exhibited improvements in the scores of CAT, mMRC, and 6MWT (*p* < 0.05), as depicted in Figure 5.

### Safety

During the 1-year period of treatment and follow-up, no significant difference existed in adverse events between two groups (*p* = 0.512, Table 4).

**Table 4:** The number of adverse events.

Group	Occurred adverse events		Un-occurred adverse events		Total	*χ* ^2^	*P*
	*n*	Percent (%)	*n*	Percent (%)			
Trial group	20	36.36	35	63.64	55	0.430	0.512
Control group	25	42.37	34	57.63	59		
Total	45	39.47	69	60.53	114		

## Discussion

Shortness of breath, chronic cough, and sputum are considered characteristic symptoms of COPD. Our research findings indicate that the combined use of TCM and western medicine reduced clinical symptoms, particularly in sputum, compared to the control group (p < 0.05). In the sixth month of the trial, the TCM granule treatment demonstrated superiority in terms of the total effective rate (*p* < 0.05), highlighting its potential in improving clinical symptoms.

According to the Global Initiative for Chronic Obstructive Lung Disease (GOLD) guidelines (2020) (1), a COPD exacerbation is defined as an acute worsening of respiratory symptoms that requires additional therapy, such as hospitalization. Our findings revealed that the combined use of TCM and western medicine reduced the frequency and duration of hospitalization compared to baseline. COPD exacerbations are complex events, often triggered by respiratory viral or bacterial infections (17). It has been reported that COPD patients with positive cultures for *P. aeruginosa* in the airways have an increased risk of exacerbations (18). This underscores the importance of infection prevention in reducing COPD exacerbations. Some studies have suggested the potential of TCM in preventing viral infections (19, 20). Unfortunately, our findings did not show a significant difference in exacerbation rates between the trial group and control group. This may be attributed to limitations in follow-up time and sample size. Additionally, we acknowledge the complexity of evaluating exacerbations, as non-infectious factors such as age, gender, and comorbidities can also contribute. A novel risk score called BODEx (Body mass index, airflow Obstruction, Dyspnea, and Exercise capacity) has been proposed to assess COPD exacerbations in relation to comorbidities (21). Incorporating comprehensive factors, including clinical parameters, comorbidities, occupation, and economic status, in future questionnaires may enhance our sensitivity in evaluating the effects of TCM on COPD.

Patients’ quality of life was assessed using the CAT questionnaire. Our research findings indicate that the combined use of TCM and western medicine led to a reduction in CAT scores, although no significant difference was observed compared to the control group. This implies that TCM did not demonstrate superiority over placebo, which may be due to the insufficient sample size. We observed a significant reduction in sputum, one of the items in the CAT, with the use of TCM. However, we did not observe a similar improvement in shortness of breath and cough, which might have contributed to the lack of overall superiority in the CAT scores. In the future, adjustments can be made to enhance the efficacy of TCM in addressing shortness of breath and cough.

Exercise tolerance was assessed using the 6MWT and mMRC. Although both measures showed improvement after treatment, the trial group did not exhibit superiority compared to the control group. It is worth noting that the 6MWT is a cost-effective and well-tolerated test, but it is highly sensitive to variations in methodology and environmental factors. On the other hand, the mMRC scale has a short completion time but only assesses breathlessness (22). Moreover, exercise tolerance is not easily modifiable, and the treatment period may have been too short. These observations indicate that a one-year observation period may not be sufficient to detect significant changes in exercise tolerance.

Lung function parameters, particularly FVC (%), FEV1 (%), and FEV1/FVC, are vital for evaluating the effectiveness of TCM granules. At baseline, significant differences were observed in FVC and FEV1 (*p* < 0.05). The Normative Aging Study has reported that FEV1 is more strongly associated with mortality than FVC in the general population (23). Additionally, another study highlighted that FEV1 is a stronger predictor of all-cause mortality than FVC in moderate COPD patients with an elevated cardiovascular risk (24). Based on our research, during month 6 and month 12, both FEV1 (*p* < 0.001, *p* = 0.003) and FEV1% (p < 0.001, *p* = 0.029) significantly improved in the trial group compared to baseline. Furthermore, during month 6 and month 12, the D-value of FEV1 in the trial group exhibited a significant difference compared to the control group (*p* = 0.046, *p* = 0.013), suggesting that the combined use of TCM and western medicine achieved a sustained superior effect compared to monotherapy with western medicine.

Additionally, the D-value of FVC% also exhibited a significant difference compared to the control group, revealing the superiority of the co-application of TCM and western medicine. Previous research has indicated a strong positive correlation between peak oxygen uptake (VO2peak) (% pred.) and FEV1 (% pred.) (25). This suggests that FEV1% may potentially reflect a patient’s maximum aerobic capacity. During month 6, both FVC and FEV1% in the trial group showed a significant difference compared to baseline, suggesting that the co-application of TCM and western medicine may increase patients’ maximum aerobic capacity after treatment. However, further investigation is needed to verify this relationship in the future. Furthermore, during month 12, the control group exhibited an obvious decline in FEV1/FVC compared to baseline, while the trial group showed no significant difference in comparison to baseline. This may imply that the combination of TCM and western medicine could suppress the deterioration of FEV1/FVC. Overall, TCM granules, when used in combination with western medicine, demonstrate potential in improving lung function.

Regarding adverse events, no significant differences were observed between the trial group and control group, indicating the safety of applying TCM clinically.

There are several limitations to our trial. The final number of included cases was smaller than the estimated sample size based on efficiency in the previous stage, which may introduce bias into our research results. However, considering the superiority of the total effective rate in the trial group during month 6, our research results are reliable and support the research hypothesis. Second, this trial was concluded in 2015, and the data may be considered “out-of-date.” However, we obtained similar conclusions to a meta-analysis published in 2021, such as alleviating clinical symptoms, reducing exacerbation frequency, improving quality of life, and demonstrating clinical efficacy (26). Thus, our trial still provides valuable evidence to verify the effectiveness of TCM in clinical practice.

## Conclusion

In this study, we evaluated the efficacy of TCM in various aspects, including clinical symptoms, lung function, hospitalization, quality of life, and safety. The results demonstrate that the combination of western medicine and TCM is superior to western medicine monotherapy in stable COPD patients, particularly in terms of sputum symptoms, D-value of FVC%, and FEV1. This indicates the potential for further exploration of TCM in the treatment of COPD.

## Data availability statement

The original contributions presented in the study are included in the article/supplementary material, further inquiries can be directed to the corresponding authors.

## Ethics statement

The studies involving humans were approved by the Ethics Committee of the First Affiliated Hospital of Zhejiang Chinese Medical University, with the approval identifier 2012-K-001-02. Trial registration number: ChiCTR-TRC-13003531, registered on August 29, 2013. The studies were conducted in accordance with the local legislation and institutional requirements. The participants provided their written informed consent to participate in this study.

## Author contributions

RC: Writing – original draft, Data curation. KN: Writing – original draft, Visualization. CJ: Writing – review & editing, Methodology. ZL: Writing – review & editing, Investigation. YY: Writing – review & editing, Investigation. GL: Writing – review & editing, Investigation. JY: Writing – review & editing, Data curation. ZW: Writing – review & editing, Funding acquisition, Conceptualization.

## References

[1] Global Initiative for Chronic Obstructive Pulmonary Disease. (2020). Available at: https://goldcopd.org (Accessed December 03, 2019).

[2] Jemal A Ward E Hao Y Thun M. Trends in the leading causes of death in the United States, 1970-2002. JAMA. (2005) 294:1255–9. doi: 10.1001/jama.294.10.1255, PMID: 16160134

[3] Lozano R Naghavi M Foreman K Lim S Shibuya K Aboyans V. Global and regional mortality from 235 causes of death for 20 age groups in 1990 and 2010: a systematic analysis for the global burden of disease study 2010. Lancet. (2012) 380:2095–128. doi: 10.1016/s0140-6736(12)61728-0, PMID: 23245604 PMC10790329

[4] Vos T Flaxman AD Naghavi M Lozano R Michaud C Ezzati M. Years lived with disability (Ylds) for 1160 sequelae of 289 diseases and injuries 1990-2010: a systematic analysis for the global burden of disease study 2010. Lancet. (2012) 380:2163–96. doi: 10.1016/s0140-6736(12)61729-2 23245607 PMC6350784

[5] World Health Organization. The top 10 causes of death. Available at: https://wwwwhoint (Accessed December 09, 2020).

[6] Wang C Xu J Yang L Xu Y Zhang X Bai C. Prevalence and risk factors of chronic obstructive pulmonary disease in China (the China pulmonary health [Cph] study): a National Cross-Sectional Study. Lancet. (2018) 391:1706–17. doi: 10.1016/s0140-6736(18)30841-9 29650248

[7] Global Initiative for Chronic Obstructive Pulmonary Disease. Available at: https://goldcopd.org (Accessed December 14, 2009).

[8] Keating GM. Tiotropium bromide inhalation powder: a review of its use in the Management of Chronic Obstructive Pulmonary Disease. Drugs. (2012) 72:273–300. doi: 10.2165/11208620-000000000-00000 22217233

[9] Wang M Li J Li S Xie Y. Effects of comprehensive therapy based on traditional Chinese medicine patterns on older patients with chronic obstructive pulmonary disease: a subgroup analysis from a four-Center, randomized, controlled study. Front Med. (2014) 8:368–75. doi: 10.1007/s11684-014-0360-0, PMID: 25204290

[10] Peng JQ Ye WX Fan FC. The effect of Jinshui Liujun decoction on clinical symptoms and inflammatory response in patients with severe and extremely severe chronic obstructive pulmonary disease. Chin J Gerontol. (2020) 40:2759–62.

[11] Huang X Duan X Wang K Wu J Zhang X. Shengmai injection as an adjunctive therapy for the treatment of chronic obstructive pulmonary disease: a systematic review and meta-analysis. Complement Ther Med. (2019) 43:140–7. doi: 10.1016/j.ctim.2019.01.020, PMID: 30935521

[12] Yue QF Chen YQ. Fan XS a study on the mechanism of action of Jinshui Liujun decoction in the treatment of chronic obstructive pulmonary disease based on network pharmacology. J Nanjing Univ Trad Chin Med. (2020) 36:358–64. doi: 10.27253/d.cnki.gnjzu.2020.000671

[13] Ritchie AI Wedzicha JA. Definition, causes, pathogenesis, and consequences of chronic obstructive pulmonary disease exacerbations. Clin Chest Med. (2020) 41:421–38. doi: 10.1016/j.ccm.2020.06.007, PMID: 32800196 PMC7423341

[14] Hsu KY Lin JR Lin MS Chen W Chen YJ Yan YH. The modified Medical Research Council dyspnoea scale is a good indicator of health-related quality of life in patients with chronic obstructive pulmonary disease. Singapore Med J. (2013) 54:321–7. doi: 10.11622/smedj.2013125, PMID: 23820542

[15] Dajczman E Wardini R Kasymjanova G Préfontaine D Baltzan MA Wolkove N. Six minute walk distance is a predictor of survival in patients with chronic obstructive pulmonary disease undergoing pulmonary rehabilitation. Can Respir J. (2015) 22:225–9. doi: 10.1155/2015/280187, PMID: 26252533 PMC4530856

[16] Okutan O Tas D Demirer E Kartaloglu Z. Evaluation of quality of life with the chronic obstructive pulmonary disease assessment test in chronic obstructive pulmonary disease and the effect of Dyspnea on disease-specific quality of life in these patients. Yonsei Med J. (2013) 54:1214–9. doi: 10.3349/ymj.2013.54.5.1214, PMID: 23918572 PMC3743182

[17] Wedzicha JA Singh R Mackay AJ. Acute Copd Exacerbations. Clin Chest Med. (2014) 35:157–63. doi: 10.1016/j.ccm.2013.11.001 24507843

[18] Eklöf J Sørensen R Ingebrigtsen TS Sivapalan P Achir I Boel JB. Pseudomonas aeruginosa and risk of death and exacerbations in patients with chronic obstructive pulmonary disease: An observational cohort study of 22 053 patients. Clinical Microb Infection. (2020) 26:227–34. doi: 10.1016/j.cmi.2019.06.011, PMID: 31238116

[19] Zeng Y Jiang F Chen Y Chen P Cai S. Exercise assessments and trainings of pulmonary rehabilitation in COPD: a literature review. Int J Chron Obstruct Pulmon Dis. (2018) 13:2013–23. doi: 10.2147/copd.S167098, PMID: 29983556 PMC6027710

[20] Weiss ST Segal MR Sparrow D Wager C. Relation of Fev1 and peripheral blood leukocyte count to Total mortality. The normative aging study. Am J Epidemiol. (1995) 142:493–8. doi: 10.1093/oxfordjournals.aje.a117665, PMID: 7677128

[21] Ge H Liu X Gu W Feng X Zhang F Han F. Distribution of Copd comorbidities and creation of acute exacerbation risk score: results from Scicp. J Inflamm Res. (2021) 14:3335–48. doi: 10.2147/jir.S315600, PMID: 34290518 PMC8289369

[22] Lin LL Shan JJ Xie T Xu JY Shen CS Di LQ. Application of traditional Chinese medical herbs in prevention and treatment of respiratory syncytial virus. Evid Based Complement Alternat Med. (2016) 2016:6082729–13. doi: 10.1155/2016/6082729, PMID: 27688789 PMC5027054

[23] Wang X Liu Z. Prevention and treatment of viral respiratory infections by traditional Chinese herbs. Chin Med J. (2014) 127:1344–50. doi: 10.3760/cma.j.issn.0366-6999.20132029 24709192

[24] Bikov A Lange P Anderson JA Brook RD Calverley PMA Celli BR. FEV (1) is a stronger mortality predictor than FVC in patients with moderate COPD and with an increased risk for cardiovascular disease. Int J Chron Obstruct Pulmon Dis. (2020) 15:1135–42. doi: 10.2147/copd.S242809, PMID: 32547001 PMC7247606

[25] Carvalho-Jr LCS Trimer R Arêas GP Caruso FC Zangrando KT Jürgensen SP. COPD assessment test and FEV (1): do they predict oxygen uptake in COPD? Int J Chron Obstruct Pulmon Dis. (2018) 13:3149–56. doi: 10.2147/copd.S167369, PMID: 30349223 PMC6183695

[26] Xiong C Li Y Zhuang G Zeng Y Wei H Li C. Clinical efficacy and safety of Chinese herbal medicine versus placebo for the treatment of chronic obstructive pulmonary disease: a systematic review and meta-analysis. Complement Ther Med. (2021) 59:102691. doi: 10.1016/j.ctim.2021.102691, PMID: 33618010

